# Do the math: modernizing the FDA’s evidence paradigm for precision medicine

**DOI:** 10.1172/JCI211143

**Published:** 2026-08-17

**Authors:** Peter J. Pitts, Judea Pearl, Arthur L. Caplan, Robert Goldberg

**Affiliations:** 1 Center for Medicine in the Public Interest, New York, New York, USA.; 2 University of Paris School of Medicine, Paris, France.; 3 UCLA, Los Angeles, California, USA.; 4 NYU Grossman School of Medicine (emeritus), New York, New York, USA.

## The arithmetic problem

Rare disease medicine poses a fundamental challenge. While traditional clinical trials were designed to estimate average treatment effects across large populations, rare disease populations often number in the hundreds, dozens, or even single digits. In this context, the question is not whether a treatment works on average but whether it works for a particular patient. The FDA’s greatest challenge over the next decade may not be artificial intelligence (AI), gene editing, cell therapy, or advanced manufacturing. It may be arithmetic.

For most of the modern era, uncertainty in drug development has been addressed the same way: generate more evidence. Need greater confidence? More statistical power? Stronger evidence? Enroll more patients. Initially, scaling up served medicine remarkably well. It was foundational for shaping the modern pharmaceutical enterprise and remains indispensable when large, well-controlled trials are feasible, ethical, and informative. The problem is that medicine is evolving.

As biology becomes more precise, patient populations become smaller. Diseases once treated as single conditions are increasingly divided into molecularly distinct subgroups and subtypes. A subtype can be further distinguished by specific mutations present in only a handful of patients. For instance, in amyotrophic lateral sclerosis (ALS), what was once viewed as a single clinical syndrome is increasingly being subdivided into biologically distinct populations, including genetically defined forms that may respond differently to targeted therapies (1). The more precisely disease biology is defined, the smaller the relevant patient population often becomes. Precision can increase confidence in mechanism while simultaneously reducing the feasibility of conventional clinical trials. We have adopted the phrase “denominator collapse” to describe this phenomenon (2), which has become one of the defining realities of modern drug development.

Moreover, across rare and genetic diseases, scientists increasingly understand the molecular events that drive disease progression. In Duchenne muscular dystrophy (DMD), for example, therapeutic approaches such as exon skipping are designed to directly address very specific disease-causing mutations that disrupt dystrophin production, in some cases only touching less than 10% of all patients with DMD (3, 4). Biological knowledge is advancing faster than regulatory science. The challenge for this era of medicine is generating conventional clinical evidence before the disease causes irreversible harm.

The future of medicine is increasingly being built for populations too small to support evidentiary models inherited from the twentieth century. At some point, “enroll more patients” stops being a scientific strategy and for rare diseases, becomes a show-stopper since there are not more patients. Patients, physicians, regulators, and developers need to know not merely whether a treatment appears beneficial for a group of people with a shared disease, but for whom it works, for whom it fails, and for whom it may be dangerous.

**Figure 1:**
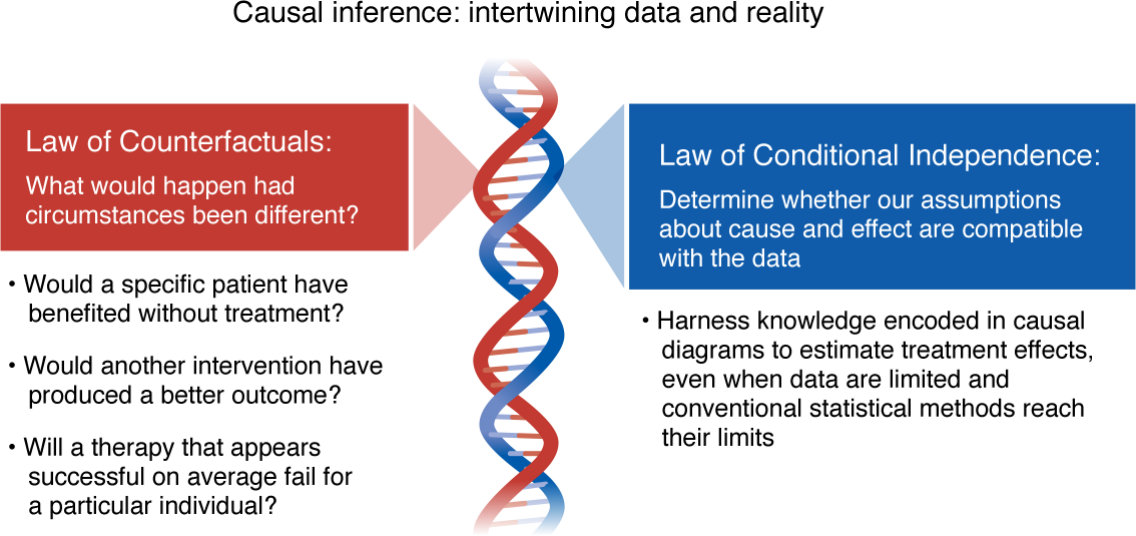
Causal inference: the double helix for personalized medicine in rare disease. Causal inference can be conceptualized as a “double helix of causal thinking,” intertwining data and reality through two fundamental principles. The first strand is the Law of Counterfactuals: What would have happened had circumstances been different? In rare disease, this means asking whether a specific patient would have benefited without treatment, whether another intervention would have produced a better outcome, or whether a therapy that appears successful on average may fail for a particular individual. The second strand is the Law of Conditional Independence: How can we determine whether our assumptions about cause and effect are reflected in the data? This principle enables researchers to use causal diagrams and structural models to identify treatment effects, even when data are limited and conventional statistical methods reach their limits. Together, these principles offer a path toward faster, less expensive, and more precise drug development by combining randomized and observational evidence, identifying likely responders before approval, leveraging real-world data more effectively, and estimating individual treatment effects rather than relying solely on population averages. Causal inference is an essential step beyond the Plausible Mechanism Framework (5) response to precision medicine regulation. The randomized clinical trial remains indispensable, but without causal inference, it remains largely confined to providing answers based on averages of large populations. Rare disease and personalized medicine requires the ability to reason about causes, interventions, and counterfactual outcomes. In that sense, causal inference is not merely another analytical tool. It is the scientific framework that can connect data to reality and make truly individualized treatment decisions possible.

The FDA’s Plausible Mechanism Framework (5) represents an important institutional response to the precision medicine era by recognizing that biological understanding itself can carry evidentiary value. Yet plausible mechanisms are only a starting point. Causal inference offers a pathway from plausible mechanisms to causal mechanisms, providing rigorous methods for integrating randomized and real-world evidence, supporting external controls and natural-history comparisons, identifying patient-level treatment effects, and enabling responsible generalization across related diseases and platform technologies (Figure 1).

These concepts align closely with the FDA’s modernization initiatives and the upcoming Prescription Drug User Fee Act (PDUFA VIII) negotiations. Better methods for evaluating causal evidence can reduce regulatory uncertainty, lower the cost of capital investment, and expand investment in therapies for rare and devastating diseases. Ultimately, the future of precision medicine will depend not on enrolling more patients, but on learning more from the patients we have. Here, we discuss how causal inference, denominator collapse, patient demand, and FDA modernization can reshape biopharmaceutical development and regulatory science.

## Denominator collapse and the limits of scale

Randomized clinical trials remain a powerful tool for estimating causal effects and building regulatory confidence. Larger trials enrolling more subjects produce narrower confidence intervals and greater statistical power. Additional studies reduce uncertainty. Randomization minimizes bias and creates strong counterfactual comparisons. But randomized trials are tools designed to generate evidence for common diseases. When diseases affect small numbers of patients, a different tool is needed. Precision medicine requires a transition from evidence generated through scale to evidence generated through causation (6).

This is not a temporary problem confined to rare diseases. Oncology, neurology, inherited metabolic disorders, gene therapies, antisense technologies, RNA-based medicines, and individualized therapies are all moving toward increasingly precise molecularly aided definitions. When patient populations collapse from thousands to dozens, insisting on evidence that cannot realistically be generated is not scientific rigor. Evidentiary standards should remain rigorous, but they must evolve alongside the biology they are intended to regulate.

Bayesian methods incorporating historical controls, disease registries, and other real-world evidence have become increasingly valuable in addressing the limitations of small datasets, making rare disease drug development more efficient. FDA initiatives such as the Complex Innovative Trial Design Program (7) and the Bayesian Supplemental Analysis Demonstration Project (8) reflect growing recognition of their value. Yet Bayesian analysis cannot fully answer the questions that personalized medicine demands. Bayesian methods ask: What is the probability of observing these data under a given hypothesis? Causal inference asks a more fundamental question: What would happen if we intervened? (Figure 1).

## FDA’s Plausible Mechanism Framework

The FDA’s Plausible Mechanism Framework (5) permits regulatory confidence to be supported by biological plausibility, mechanistic understanding, target engagement, biomarker response, and natural-history evidence even before traditional demonstrations of clinical benefit are complete. This reflects an increasingly important reality; biological understanding itself has evidentiary value. The Plausible Mechanism Framework is important not because it creates a pathway around conventional evidence, but because it recognizes that the nature of evidence itself is changing.

The FDA’s embrace of plausible-mechanism thinking can be understood as an institutional response to denominator collapse. As patient populations become smaller and more genetically defined, scientific understanding must shoulder a larger share of evidentiary weight.

## From plausible mechanisms to causal mechanisms

Plausibility asks whether a biological story makes sense. Causality asks whether available evidence supports the conclusion that an intervention produced an observed outcome. The FDA’s framework establishes an important foundation by recognizing mechanistic plausibility. The next step is to develop rigorous approaches for evaluating causal mechanisms. This is where causal inference becomes essential.

The intellectual foundation for this transition was established through the work of Judea Pearl and further advanced by Miguel Hernán, James Robins, and others (9). Rather than simply asking whether treated patients appear to do better than untreated patients, causal inference asks deeper questions: Did treatment cause the observed outcome? What would have happened otherwise? Through which biological pathways did benefit occur? Would similar effects occur in related populations?

Critics sometimes portray causal inference, external controls, natural-history comparators, and real-world evidence as attempts to lower evidentiary standards (10). That criticism misunderstands the challenge. Causal inference does not replace scientific discipline; rather, it formalizes it. By making assumptions explicit, testing alternative explanations, quantifying uncertainty, and constructing transparent counterfactuals, causal methods often force investigators to confront questions that traditional analyses leave implicit.

The alternative to a large, randomized trial is rarely an inferior substitute. More often, it is an opportunity to learn without conducting impossible or unethical experiments. For instance, researchers should not be expected to randomize children with rapidly progressive fatal diseases to prolonged placebo exposure when disease biology is understood, and the intervention plausibly addresses the underlying defect (11, 12). In this context, the ethical obligation is to ask what form of rigor is appropriate when delayed treatment carries its own measurable harm (13, 14).

Thus, causal inference is not a shortcut to generating less evidence. Instead, it facilitates a more complete accounting of what the available evidence can and cannot tell us. Causal inference represents a different form of rigor — one increasingly suited to the arithmetic of precision medicine.

## Causal inference, regulatory learning, and FDA modernization

In its adoption of the Plausible Mechanism Framework, the FDA will increasingly evaluate therapies based on shared biological mechanisms rather than treating every disease as an entirely separate evidentiary universe. The central question is no longer whether evidence can be generated for a single therapy. It is whether knowledge generated for one therapy can responsibly inform the next.

The FDA modernization is often framed in technological terms — AI, computational toxicology, advanced manufacturing, digital health tools, and alternatives to animal testing. Those technologies matter, as do platform technologies, master protocols, Bayesian borrowing, self-controlled studies, representative natural-history databases, and cumulative evidence generation, which create opportunities for regulatory learning (15). But FDA modernization is fundamentally a challenge to generate evidence that supports regulatory decision making, not a challenge to develop technology per se.

Causal inference is the bridge between the information technology provides and better regulatory decision making. Real-world evidence can generate observations, and humans and AI can identify patterns, but causal inference helps determine whether those observations and patterns are informative. While AI and other technologies may help the FDA process information more efficiently, efficiency alone is not a substitute for scientific judgment.

## Causal inference as an economic tool

Scientific uncertainty becomes regulatory uncertainty, which can lead to financial uncertainty. Financial uncertainty ultimately increases the cost of capital. Viewed through that lens, causal inference is not merely a statistical tool. It is an economic tool.

Evidence to support causal inference derives from many steps along the drug development path. This path includes demonstrating a therapeutic agent’s target engagement, establishing persuasive natural-history comparators, validating biologically relevant biomarkers, and constructing a rigorous narrative. Beyond a compelling use case, these steps each reduce uncertainty. The more predictable the FDA becomes in evaluating causal evidence, external controls, platform approaches, and real-world evidence, the lower that risk premium becomes. Lower risk attracts investment and expands the range of diseases that become economically viable as development targets (16).

## What should PDUFA VIII fund?

First signed into law in 1992, PDUFA establishes a funding source to support the FDA and facilitate efficient review of new drug applications. PDUFA can also fund reform initiatives, including pilot programs and investment in new technology and scientific updates. The PDUFA program collects fees for submission of most types of new applications. Periodic reauthorization of PDUFA involves negotiation between the FDA and representatives from the pharmaceutical industry, and it requires approval by Congress.

The negotiation of PDUFA VIII offers an opportunity to invest not only in the speed afforded by new technology, but in the scientific judgment needed to evaluate new categories of evidence consistently and credibly. PDUFA VIII should not merely fund review capacity. It should also fund intellectual capacity (17). If causal inference is going to play a larger role in evaluating therapies for increasingly small and biologically defined populations, the FDA must develop a knowledgeable workforce and provide training, as well as the institutional infrastructure necessary to apply those methods predictably, transparently, and credibly.

The FDA already possesses world-class expertise in biostatistics, clinical pharmacology, and regulatory review. The next step is ensuring comparable biomedical expertise to support causal inference and modern evidence-generation methods (17). If the FDA is expected to evaluate twenty-first-century science using twentieth-century evidentiary infrastructure, patients, sponsors, and payers will bear the cost.

## A regulatory science agenda for precision medicine

The FDA’s present challenge is determining how to generate reliable knowledge when biology increasingly fragments diseases into ever-smaller populations. Judea Pearl and others provided the mathematical framework in the form of Bayesian methods. Denominator collapse explains why those tools are becoming indispensable. The FDA’s Plausible Mechanism Framework (5) demonstrates how regulatory policy is beginning to respond.

The PDUFA VIII negotiations offer an opportunity to transform those concepts into institutional capability (17). Precision medicine is already dividing diseases into increasingly narrow biological categories. If the FDA is to evaluate causal evidence consistently and credibly, it must develop the knowledge and regulatory culture necessary to assess evolving forms of evidence with the same rigor historically applied to traditional clinical trials. For patients with DMD, Huntington disease, ALS, and countless other serious disorders, failure to evolve with the evidence will be experienced in lost function, lost time, and lost opportunity.

The future of precision medicine will not be determined by our ability to enroll more patients. In many diseases, those patients simply do not exist. Success will depend on our ability to learn more from the patients we have. Modernizing the FDA’s decision-making process is not regulatory leniency. It is scientific realism — and it’s time to do the math.

## About the authors

PJP is President of the Center for Medicine in the Public Interest and Former Associate Commissioner of the US FDA. JP is Chancellor’s Professor of Computer Science and Statistics at UCLA and a Turing Prize Laureate. AC is Professor Emeritus at the NYU Grossman School of Medicine. RG is Vice President of the Center for Medicine in the Public Interest.

## Conflict of interest

PJP is a board member at Neurocentryx and Brainstorm Therapeutics. AC is a WebMD Bioethics Commentator and serves on the data and safety monitoring boards of Otsuka and iEcure.

## Version 1 08/17/2026

Electronic publication
